# Can patients with good tumor regression grading after neoadjuvant chemoradiotherapy be exempted from lateral lymph node dissection?

**DOI:** 10.1007/s12672-022-00607-w

**Published:** 2022-12-30

**Authors:** Xianwei Liu, Xuyang Yang, Qingbin Wu, Tong Zhang, Dan Jiang, Ziqiang Wang

**Affiliations:** 1 Colorectal Cancer Center, Department of General Surgery, West China Hospital, Sichuan University, Chengdu, China; 2 Department of General Surgery, Jiujiang NO.1 People’s Hospital, Jiujiang, Jiangxi China; 3 Department of Imaging Center, West China Hospital, Sichuan University, Chengdu, China; 4 Department of Pathology, West China Hospital, Sichuan University, Chengdu, China; 5 Department of Gastrointestinal Surgery, West China Hospital, Sichuan University, No. 37 Guo Xue Alley, Chengdu, 610041 Sichuan China

**Keywords:** Advanced rectal cancer, Neoadjuvant chemoradiotherapy, Lateral lymph node metastasis, Tumor regression grading

## Abstract

**Objective:**

To investigate whether lateral lymph node (LLN) dissection (LLND) can be exempted in patients with good tumor regression grading (TRG) after neoadjuvant chemoradiotherapy (nCRT)?

**Methods:**

A retrospective study was conducted on consecutive patients with advanced rectal cancer who underwent nCRT and total mesorectal resection plus selective LLND at our institution. The primary outcomes are the relationship between LLN metastasis (LLNM) and magnetic resonance imaging TRG (mrTRG) and the relationship between LLNM and pathological TRG (pTRG).

**Results:**

A total of 91 patients were included, of which 24 patients (26.4%) had LLNM, 67 patients (73.6%) had no LLNM. There were significant differences of the maximum short-axis of LLN before and after nCRT, short-axis reduction rate of the LLN with maximum short-axis, length diameter reduction rate of primary tumor, mrTRG, and pTRG between the two groups. Multivariate logistic regression showed that mrTRG (*P* = 0.026) and pTRG (*P* = 0.013) were independent predictors for LLNM. The combination used by mrTRG and the maximum short-axis of LLNs ≥ 8 mm before nCRT and the maximum short-axis of LLN ≥ 5 mm after nCRT achieved specificity of 0.970, positive predictive value (PPV) of 0.867, and negative predictive value (NPV) of 0.855. The same combination used by pTRG achieved the specificity of 0.970, PPV of 0.857 and NPV of 0.844.

**Conclusion:**

The suspected positive LLNs tend to be sterilized by nCRT in patients who have a very good response to nCRT. It is rational to avoid LLND in patients whose primary tumor and LLNs both show good response to nCRT.

**Supplementary Information:**

The online version contains supplementary material available at 10.1007/s12672-022-00607-w.

## Introduction

The treatment of advanced rectal cancer patients with suspected positive lateral lymph node (LLN) remains controversial between Eastern and Western scholars [1, 2]. In West, neoadjuvant chemoradiotherapy (nCRT) combined with total mesorectal excision (TME) is the standard treatment [3]. However, the standard treatment method in East represented by Japan is TME with prophylactic lateral lymph node (LN) dissection (pLLND) [4]. In China, based on the results of Akiyoshi et al. [5], selective lateral lymph node dissection (sLLND) is recommended if suspected positive LLN is detected on pre-treatment imaging, and pLLND is not performed if there is no suspected positive LLN [6].

Recently, there were some studies [7, 8] reported that TME with sLLND after nCRT was more beneficial to reduce lateral local recurrence (LLR) and improve overall survival (OS) for advanced rectal cancer patients with suspected lateral lymph node metastasis (LLNM). Therefore, both Western and Eastern scholars have gradually become interested in TME plus sLLND after nCRT for such diseases [9]. However, there is no consensus on what kinds of patients should undergo sLLND after nCRT and what kinds should be exempted. Therefore, the clinical management of such patients requires more rigorous and optimal evaluation.

In previous reports, pathological tumor regression grading (pTRG) have been established as an important factor for the survival outcomes of patients with advanced rectal cancer after nCRT and also confirmed to be closely associated with mesorectal LN (MLN) regression grading (LRG) [10–13]. TRG and LRG showed the same trend in esophageal [14] and breast [15] cancer as well as rectal cancer. Moreover, a new magnetic resonance imaging (MRI) TRG (mrTRG) scheme for advanced rectal cancer was developed by Peng et al. [16], which had good agreement with pTRG classification scheme. Therefore, we hypothesized that the regression of LLN was also correlated with pTRG and mrTRG in advanced rectal cancer patients after nCRT, which was worthy of further investigation.

We designed this retrospective study to evaluate the relationship between LLNM and pTRG and mrTRG in advanced rectal cancer patients with suspected LLNM after nCRT. It aimed to guide decision-making of clinical sLLND and the further treatment of patients with suspected LLNM but without LLND.

## Materials and methods

### Study population

This single-center cohort study was ethically approved by our institutional ethics committee and exempted from informed consent since it was retrospective.

Consecutive patients with advanced rectal cancer and suspected LLNM in our hospital who underwent TME plus sLLND after nCRT between December 2014 and June 2022 were retrospectively collected, and the data were extracted from a prospectively maintained database. The eligible criteria were as follows: rectal adenocarcinoma patients with suspected LLNM who underwent TME plus sLLND after nCRT. Patients with the following conditions were excluded: (1) multiple primary cancers; (2) recurrent rectal cancer; (3) previously treated for other cancers; (4) incomplete clinical data; (5) Stage IV patients. Pathological outcomes were used as the gold standard.

Node-to-node correspondence between preoperative and postoperative LLN on MRI image and pathology was achieved according to an ongoing clinical trial (U.S. clinical trial registration platform registration: NCT03826862). The specific experimental method was also described in our previous literature [17] (Figure S1).

### Treatment

**Fig. 1:**
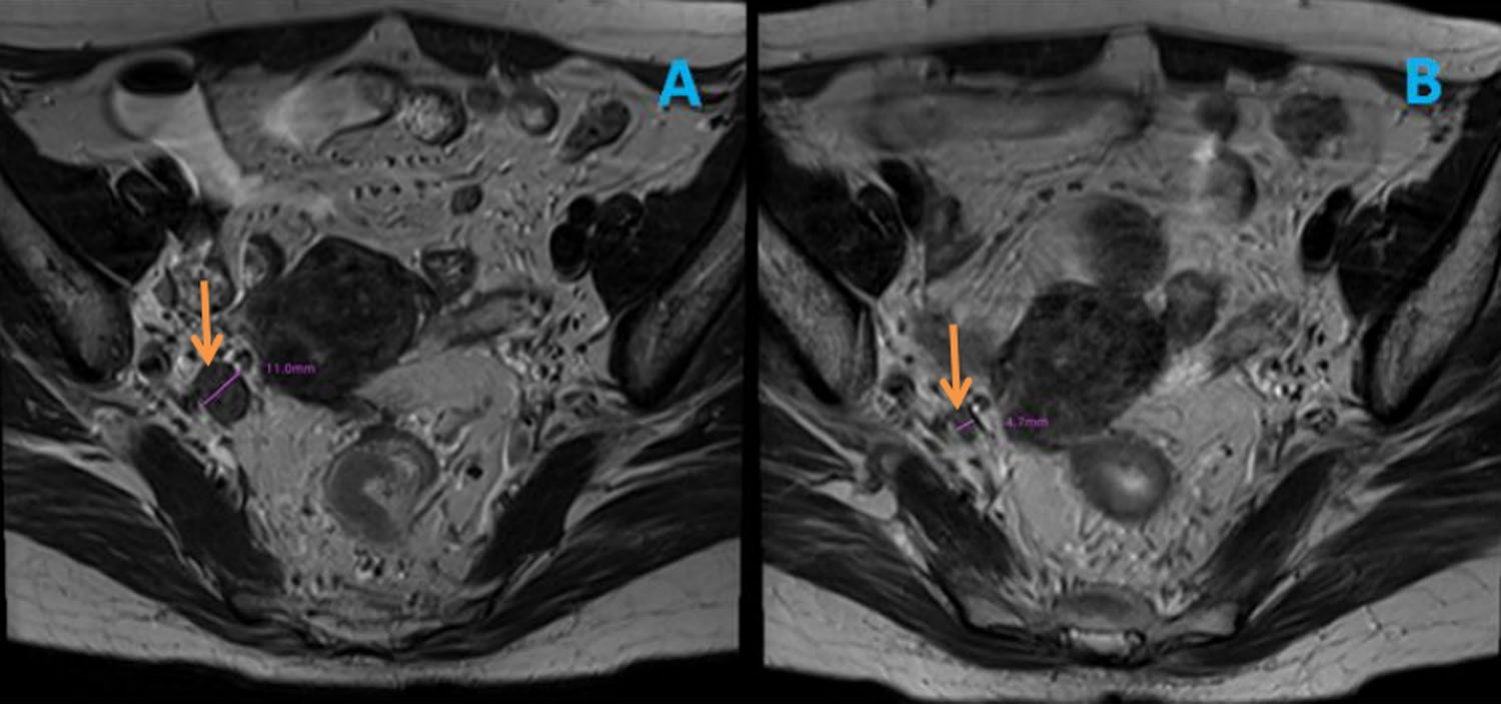
The image of suspected positive LLN and the measurement of the maximum short-axis before and after nCRT. *nCRT* neoadjuvant chemoradiotherapy; *LLN* lateral lymph node. **A** The yellow arrow points to a suspected positive lateral lymph nodes in internal iliac before long-term neoadjuvant chemoradiotherapy with a maximum short-axis of 11.0 mm. **B** The yellow arrow points to the images corresponding to the LLN in Figure A after long-term neoadjuvant chemoradiotherapy, with a maximum short-axis of 4.7 mm

The pretreatment clinical staging was mainly based on physical examination, laboratory examination, rectal ultrasound, computed tomography (CT) scan and MRI. T staging and N staging were divided according to the American Joint Committee on Cancer (AJCC) 8th staging system. The criteria of nCRT for patients with advanced rectal cancer referred to Chinese expert consensus on the diagnosis and treatment for lateral lymph node metastasis of rectal cancer [6]. The nCRT regimen was long-course CRT (45–50.4 Gy) based on 5-FU, or short-course CRT (25 Gy), and the radiotherapy area covered the lateral area. Surgery was performed 6–8 weeks after nCRT. The classification of LLN tiers referred to the Japanese guidelines [1]. Standard TME with sLLND would be performed as long as the suspected positive LLN before nCRT was still visible on imaging after nCRT (no matter how small the regression was) (Fig. 1) [18, 19]. The extent of LLND: LNs in internal iliac (263d, 263p) and obturator (283) areas were routinely dissected, while LNs in external iliac or common iliac areas was dissected only when there was suspected positive LN in these two areas. Bilateral LLND should only be considered when there were suspected positive LLN in both pelvic sidewalls. Postoperative adjuvant chemotherapy was only given to the patients with high risk factors according to pathological outcomes.

### MRI assessment

**Fig. 2:**
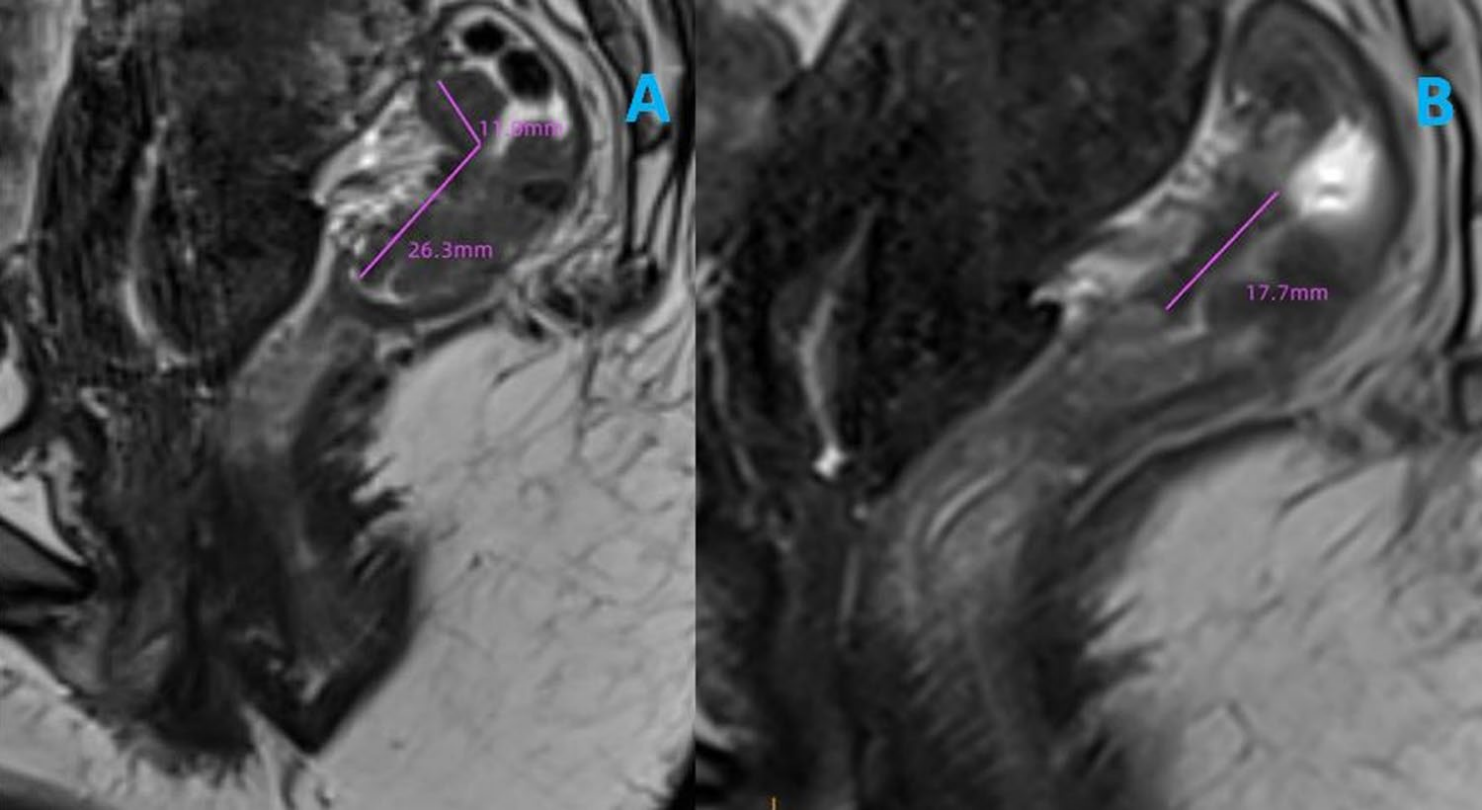
The measurement of the maximum length diameter of tumor before and after nCRT. nCRT: nCRT: neoadjuvant chemoradiotherapy. **A** The purple line measures the length diameter of the primary rectal tumor before nCRT (37.3 mm). **B** The purple line measures the length diameter of the primary rectal tumor after nCRT (17.7 mm)

Mesoretal fascia (MRF), extramural venous invasion (EMVI), mrTRG and tumor length diameter were assessed by an associate chief (T.Z.) of radiology with 15 years of experience pelvic MRI. MRF [20] and EMVI [21] were evaluated according to previous studies (Table S1 and Table S2). The four-category mrTRG system was defined as follows [16] : mrTRG 0, where there was no remaining tumor tissue; mrTRG 1, where no more than 30% of the tumor remained; mrTRG 2, where 30 to 80% of the tumor remained and mrTRG 3, where more than 80% of the tumor remained. mrTRG 0–1 was regarded as good regression, and mrTRG 2–3 as poor regression. The length diameter reduction rate of tumor was defined as (Lpre–Lpost/Lpre) × 100%, where Lpre and Lpost are the maximum length diameter of tumor before and after nCRT, respectively (Fig. 2).

Referring to previous literature [22], MRI-positive criteria for LLN before nCRT were according to the maximum short-axis and malignant features (round, irregular borders and heterogeneity) in the MRI imaging before nCRT. The MRI-positive criteria for LLN after nCRT were according to the maximum short-axis in the MRI imaging after nCRT (Table S3). The short-axis reduction rate of LLN was defined as (Spre–Spost / Spre) × 100%, where Spre and Spost are the maximum short-axis of the LLN before and after nCRT, respectively. According to the previous literature [8, 22–24], this study selected the largest LLN as the target lymph node.

### Pathological assessment

Pathological outcomes of LLN and pTRG were judged by a deputy chief physician (D.J.) of the pathology department with more than 10 years of working experience. The four-category AJCC/CAP pTRG system was classified as follows [25, 26]: TRG 0, no residual tumor cells; TRG 1, single cells or small groups of cells; TRG 2, residual cancer with a desmoplastic response; TRG 3, no significant tumor regression. TRG 0–1 was regarded as good regression, and TRG 2–3 as poor regression [10].

### Statistical analysis

Categorical data were presented as number and percentage and were compared by Pearson's chi-square test or Fisher's exact test. When continuous variables conform to normal distribution, they were expressed as mean ± standard deviation and compared by t test; continuous variables were expressed as median sum (min–max) when non-normal distribution is used, and Wilcoxon test was used for comparison. Cohen kappa test was used to check the consistency between mrTRG and pTRG [27]. The kappa values just like as Landis and Koch classification [28] (0.00, poor agreement; 0.00–0.20, slight agreement; 0.21–0.40, fair agreement; 0.41–0.60, moderate agreement; 0.61–0.80, good agreement, and 0.81–1.00, excellent agreement). All available variables were used as independent variables, and logistic regression analysis was used to evaluate the relationships with LLNM. All data were analyzed by SPSS 23 Statistics software (version 23; IBM, Armonk, NY), and *P* < 0.05 was determined to be statistically significant. The graph was produced by the software GraphPad Prism 8.0.1.

## Results

### Patient characteristics

**Fig. 3:**
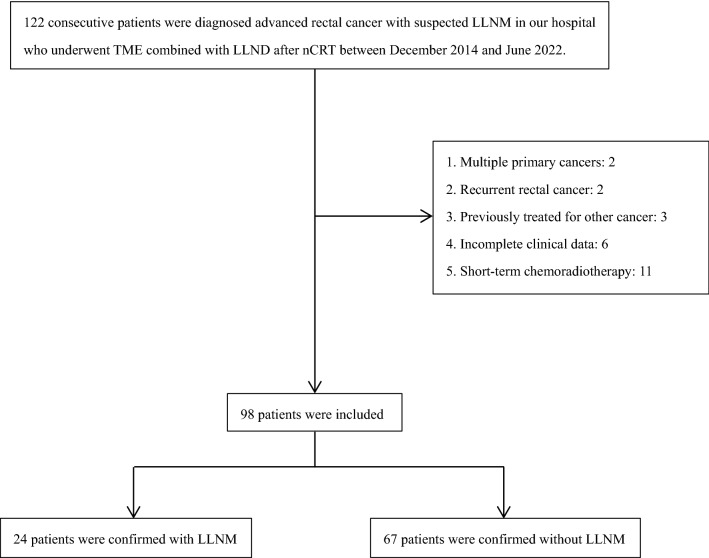
Flow chart of the study population. LLNM: lateral lymph node metastasis; *TME* total mesenteric resection; *LLND* lateral lymph node dissection; *nCRT* neoadjuvant chemoradiotherapy

There were 122 advanced rectal cancer patients with suspected positive LLN who received TME with sLLND after nCRT were included. Among them, two patients were excluded due to multiple primary cancers; two patients were excluded due to recurrent rectal cancer; three patients were excluded due to previously treated for other cancer; six patients were excluded due to incomplete clinical data; seven patients were exclude due to Stage IV; 11 patients with short-term radiotherapy were excluded due to none of them had MRI after treatment (Fig. 3).

Finally, 91 patients were included in this study, of which 24 patients (26.4%) had LLNM, 67 patients (73.6%) had no LLNM. The regional distribution of the LLN with the maximum short-axis was three in external iliac area, 40 in obturator area, and 48 in internal iliac area. Thirty-three patients were classified as mrTRG 0–1 (eight as mrTRG 0, 25 as mrTRG 1), while 58 patients were classified as mrTRG 2–3 (37 as mrTRG 2, 21 as mrTRG 3). Forty-one patients were classified as pTRG 0–1 (22 as pTRG 0, 19 as pTRG 1), while 50 patients were classified as pTRG 2–3 (42 as pTRG 2, eight as pTRG 3).The consistence between mrTRG and pTRG checked by Cohen kappa test was acceptable (к = 0.684).

The details of clinical characteristics, imaging data and pTRG were respected in Tables 1 and 2. There were no statistically differences of sex, age, distance from anal verge, pre-nCRT carcinoembryonic antigen (CEA), post-nCRT CEA, location of the LLN with the maximum short-axis, clinical T stage, clinical N stage, AJCC stage, pre-nCRT MRF, pre-NCRT EMVI, and pre-nCRT LLN status between the patients with LLNM and those without LLNM (*P* > 0.05) (Tables 1 and 2). There were statistically significant differences of the maximum short-axis of LLN before nCRT (*P* = 0.016), whether the maximum short-axis of LLN ≥ 8 mm before nCRT (*P* = 0.001), the maximum short-axis of LLN after nCRT (*P* < 0.001), whether the maximum short-axis of LLN ≥ 5 mm after nCRT (*P* < 0.001), short-axis reduction rate of the LLN with maximum short-axis (*P* = 0.001), length diameter reduction rate of primary tumor (*P* = 0.044), mrTRG (*P* = 0.004), and pTRG (*P* < 0.001) between the two groups (Table 2).

**Table 1:** The clinical characteristics of the study patients grouped by LLNM

Variables	LLNM		*P* value
	Positive (n = 24)	Negative(n = 67)	
Sex			0.611^α^
Male	14 (58.3%)	43 (64.2%)	
Female	10 (41.7%)	24 (35.8%)	
Age (year)	57.38 ± 10.55	56.13 ± 11.90	0.653^γ^
Distance from anal verge (cm)	4.08 ± 1.56	4.58 ± 1.88	0.248^γ^
Pretreatment CEA (ng/ml)	4.66 (1.48.-119)	3.23 (0.67–102)	0.161^δ^
Post-treatment CEA (ng/ml)	2.50 (0.87–100)	2.20 (0.31–16.83)	0.517^δ^
Location			0.929^β^
Obturator	11 (45.8%)	29 (43.3%)	
Internal iliac	12 (50%)	36 (53.7%)	
External iliac	1 (4.2%)	2 (3%)	

*LLNM* Lateral lymph node metastasis; *LLN* Lateral lymph node; *CEA* Carcinoembryonic antigen

^α^Pearson's chi-square test

^β^Fisher's exact test

^γ^t test

^δ^Wilcoxon test

**Table 2:** The imaging data and pTRG of the study patients grouped by LLNM

Variables	LLNM		*P* value
	Positive (n = 24)	Negative(n = 67)	
The maximum short-axis of LLN before nCRT (mm)	10.02 ± 4.34	7.67 ± 3.95	0.016^γ^
The maximum short-axis of LLN before nCRT			0.001^α^
≥ 8 mm	15 (62.5%)	16 (23.9%)	
< 8 mm	9 (37.5%)	51 (76.1%)	
Clinical T stage			0.840^β^
T2	1 (4.2%)	2 (3%)	
T3	12 (50%)	38 (56.7%)	
T4	11 (45.8%)	27 (40.3%)	
Clinical N stage			0.636^β^
N0	0 (0%)	2 (3%)	
N1	13 (54.2%)	32 (47.8%)	
N2	11 (45.8%)	33 (49.2%)	
AJCC stage			0.540^β^
II	0 (0%)	2 (3%)	
III	24 (100%)	65 (97.0%)	
Pretreatment MRF			0.269^α^
Positive	19 (62.5%)	45 (67.2%)	
Negative	5 (37.5%)	22 (32.8%)	
Pretreatment EMVI			0.429^α^
Positive	13 (54.2%)	30 (44.8%)	
Negative	11 (45.8)	37 (55.2%)	
Pretreatment LLN status			0.646^β^
MRI-positive	22 (91.7%)	61 (98.5%)	
MRI-negative	2 (8.3%)	6 (1.5%)	
The maximum short-axis of LLN after nCRT (mm)	7.84 ± 4.18	4.53 ± 3.43	< 0.001^γ^
The maximum short-axis of LLN after nCRT			< 0.001^α^
≥ 5 mm	17 (63%)	14 (20.9%)	
< 5 mm	7 (37%)	53 (79.1%)	
Short-axis reduction rate (%)	17.0 (− 11.4–66.2)	43.1 (1.0–82.4)	0.001^δ^
Length diameter reduction rate (%)	38.1 ± 16.6	48.2 ± 22.0	0.044^γ^
mrTRG			0.004^β^
mrTRG 0–1	3 (12.5%)	30 (44.8%)	
mrTRG 2–3	21 (87.5%)	37 (55.2%)	
pTRG			< 0.001^α^
pTRG 0–1	3 (12.5%)	38 (56.7%)	
pTRG 2–3	21 (87.5%)	29 (43.3%)	

*pTRG* Pathological tumor regression grading; *LLNM* Lateral lymph node metastasis; *LLN* Lateral lymph node; *nCRT* Neoadjuvant chemoradiotherapy; *MRF* Mesoretal fascia; *EMVI* Extramural venous invasion; *mrTRG* magnetic resonance imaging tumor regression grading

^α^Pearson's chi-square test

^β^Fisher's exact test

^γ^t test

^δ^Wilcoxon test

### LLNM and mrTRG

The incidence of LLNM was significantly higher in patients with mrTRG 2–3 than in patients with mrTRG 0–1 (*P* = 0.004) (Table 2). Univariate analysis showed pre-nCRT CEA (*P* = 0.074), the maximum short axis of LLN before nCRT (*P* = 0.026), whether the maximum short axis of LLN ≥ 8 mm before nCRT (*P* = 0.001), the maximum short axis of LLN after nCRT (*P* = 0.002), whether the maximum short axis of LLN ≥ 5 mm after nCRT (*P* < 0.001), short axis reduction rate of the LLN with maximum short-axis (*P* < 0.001), length diameter reduction rate of primary tumor (*P* = 0.048), mrTRG (*P* = 0.009) and pTRG (*P* = 0.001)were potential risk factors for LLNM (Table 3). Multivariate analysis (excluded pTRG since they were corrected, к = 0.684) showed only mrTRG (OR = 0.12, 95% CI: 0.02–0.78, *P* = 0.026) was an independent predictor for LLNM (Table 3). Only using mrTRG to predict LLNM achieved the sensitivity of 0.875, specificity of 0.448, positive predictive value (PPV) of 0.362, and negative predictive value (NPV) of 0.909 (Table 4). mrTRG, the maximum short-axis of LLNs ≥ 8 mm before nCRT, the maximum short-axis of LLN ≥ 5 mm after nCRT, were combined to predict LLNM. Risk score A was defined as follows: Score 1: mrTRG 2–3 AND the maximum short-axis of LLNs ≥ 8 mm before nCRT AND the maximum short-axis of LLN ≥ 5 mm after nCRT; Score 0: The rest situations. Risk score A achieved the sensitivity of 0.542, specificity of 0.970, PPV of 0.867, and NPV of 0.855 (Table 4).

**Table 3:** Logistic regression analysis of clinicopathological features and image data grouped by LLNM

Variables	Univariate analysis			Multivariate analysis (Exclude pTRG)			Multivariable analysis (Exclude mrTRG)		
	OR	95%CI	*P*	OR	95%CI	*P*	OR	95%CI	*P*
Sex	1.28	0.49–3.32	0.612						
Age	1.01	0.97–1.05	0.649						
Distance from anal verge (cm)	0.85	0.65–1.12	0.247						
Pretreatment CEA (ng/ml)	1.02	1.00–1.04	0.074	1.03	1.00–1.06	0.056	1.03	1.00–1.06	0.091
Post-treatment CEA (ng/ml)	1.09	0.97–1.22	0.168						
The maximum short-axis of LLN before nCRT (mm)	1.13	1.02–1.26	0.026	1.22	0.71–2.10	0.477	1.14	0.63–2.05	0.675
The maximum short-axis of LLN before nCRT									
≥ 8 mm	0.19	0.07–0.51	0.001	0.20	0.03–1.39	0.103	0.24	0.04–1.67	0.150
< 8 mm	1			1			1		1
Clinical T stage									
T2	1		0.840						
T3	0.63	0.05–7.59	0.717						
T4	0.82	0.07–9.93	0.872						
Pretreatment MRF	0.54	0.18–1.63	0.274						
Pretreatment EMVI	0.69	0.27–1.75	0.430						
Pretreatment LLN status	1.08	0.20–5.76	0.926						
The maximum short-axis of LLN after nCRT(mm)	1.23	1.08–1.41	0.002	0.72	0.37–1.41	0.340	0.79	0.39–1.60	0.504
The maximum short-axis of LLN after nCRT									
≥ 5 mm	0.11	0.04–0.31	< 0.001	0.40	0.07–2.45	0.323	0.30	0.05–1.87	0.198
< 5 mm	1			1		1		1	
Short-axis reduction rate	0.01	0.00–0.13	< 0.001	0.00	0.00–1.57	0.065	0.00	0.00–8.94	0.162
Length diameter reduction rate	0.09	0.01–0.98	0.048	0.71	0.02–26.19	0.851	1.03	0.02–18.88	0.091
mrTRG	0.18	0.05–0.65	0.009	0.12	0.02–0.78	0.026			
pTRG	0.11	0.03–0.40	0.001				0.12	0.02–0.64	0.013

*LLNM* Lateral lymph node metastasis; *mrTRG* Magnetic resonance imaging tumor regression grading; *pTRG* Pathological tumor regression grading; *OR* Odds ratio; *CI* Confidence interval; *CEA* Carcinoembryonic antigen; *nCRT* Neoadjuvant chemoradiotherapy; *LLN* Lateral lymph node; *MRF* Mesoretal fascia; *EMVI* Extramural venous invasion

**Table 4:** The sensitivity, specificity, PPV and NPV of mrTRG, pTRG and the maximum short-axis before and after nCRT

		LLNM		Sensitivity %	Specificity %	PPV %	NPV %
		Positive (n = 31)	Negative(n = 67)				
mrTRG	mrTRG 2–3	21	37	87.5	44.8	36.2	90.9
	mrTRG 0–1	3	30				
pTRG	pTRG 2–3	21	29	87.5	56.7	42	92.7
	pTRG 0–1	3	38				
The maximum short-axis of LLN before nCRT	≥ 8 mm	15	16	62.5	76.1	48.4	85
	< 8 mm	9	51				
The maximum short-axis of LLN after nCRT	≥ 5 mm	17	14	70.8	79.1	54.8	88.3
	< 5 mm	7	53				
Risk score A	1	13	2	54.2	97.0	86.7	85.5
	0	11	65				
Risk score B	1	12	2	50	97	85.7	84.4
	0	12	65				

Risk score A was defined as follows: Score 1:mrTRG 2–3 AND the maximum short-axis of LLNs ≥ 8 mm before nCRT AND the maximum short-axis of LLN ≥ 5 mm after nCRT; Score 0: The rest situations. Risk score B was defined as follows: Score 1: pTRG 2–3 AND the maximum short-axis of LLNs ≥ 8 mm before nCRT AND the maximum short-axis of LLN ≥ 5 mm after nCRT; Score 0: The rest situations

*PPV* Positive predictive value; *NPV* Negative predictive value; *mrTRG* Magnetic resonance imaging tumor regression grading; *pTRG* Pathological tumor regression grading; *nCRT* neoadjuvant chemoradiotherapy; *LLNM* Lateral lymph node metastasis; *LLN* Lateral lymph node

### LLNM and pTRG

The incidence of LLNM in patients with pTRG 2–3 was also significantly higher than in patients with pTRG 0–1 (*P* < 0.001) (Table 2). Univariate analyses for potential risk factors of LLNM were showed above. Multivariate analysis (excluded mrTRG since they were corrected, к = 0.684) showed only pTRG (OR = 0.11, 95% CI: 0.03–0.40, *P* = 0.001) was an independent predictor for LLNM (Table 3). Only using pTRG to predict LLNM achieved the sensitivity of 0.875, specificity of 0.567, PPV of 0.420 and NPV of 0.927 (Table 4). pTRG, the maximum short-axis of LLNs ≥ 8 mm before nCRT, the maximum short-axis of LLN ≥ 5 mm after nCRT, were combined to predict LLNM. Risk score B was defined as follows: Score 1: pTRG 2–3 AND the maximum short-axis of LLNs ≥ 8 mm before nCRT AND the maximum short-axis of LLN ≥ 5 mm after nCRT; Score 0: The rest situations. Risk score B achieved the sensitivity of 0.500, specificity of 0.970, PPV of 0.857 and NPV of 0.844 (Table 4).

### Subgroup analysis

**Fig. 4:**
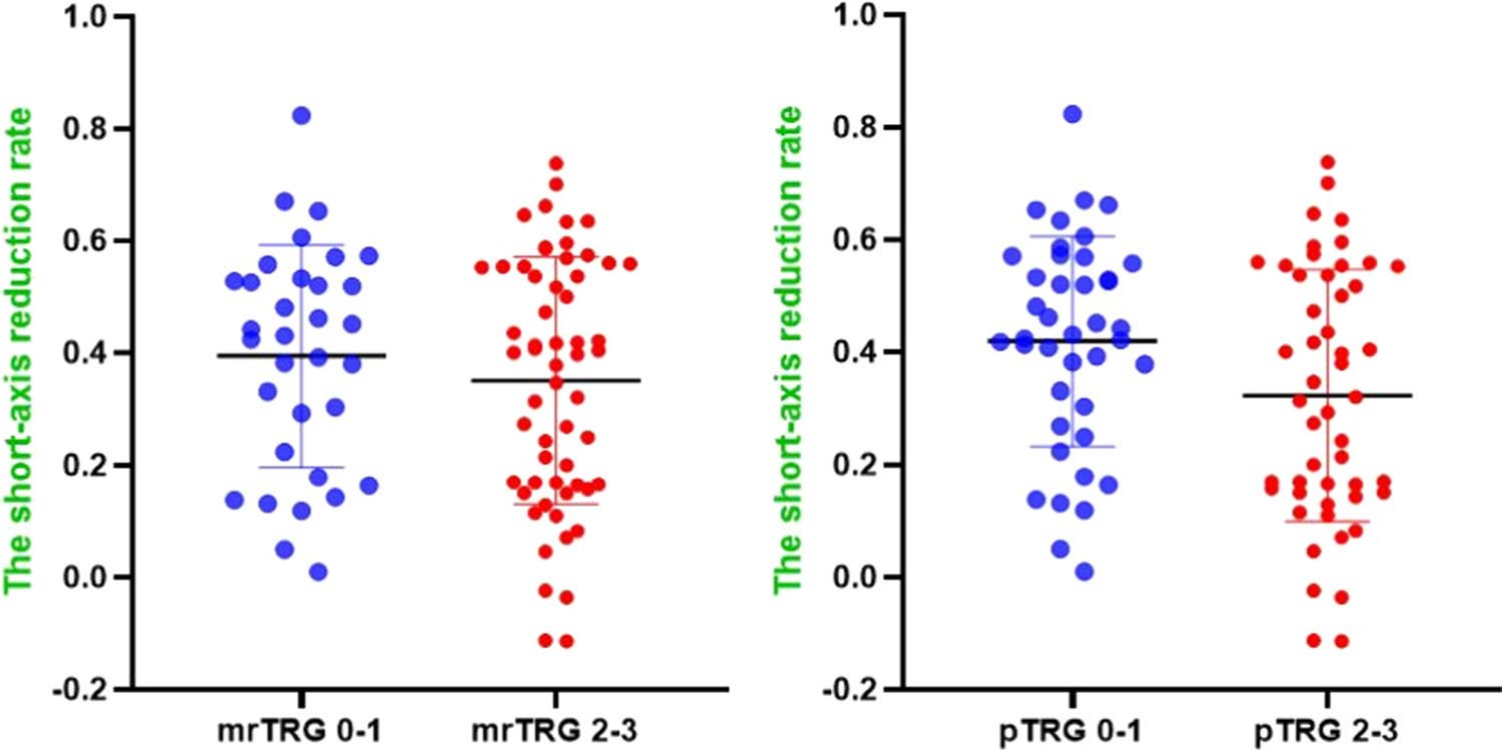
Scatter diagram of the short-axis reduction rate. *mrTRG* magnetic resonance imaging tumor regression grading; *pTRG* pathological tumor regression grading

When grouped by mrTRG, it was found that there was no difference in the maximum short-axis of LLN before and after nCRT and the short-axis reduction rate. However, when grouped by pTRG, the short-axis reduction rate in pTRG 0–1 was significantly higher than that in pTRG 2–3(*P* = 0.049) (Table 5). The scatter diagram of the short-axis reduction rate was shown in Fig. 4.

**Table 5:** The maximum short-axis of LLN before and after nCRT grouped by mrTRG and pTRG

Variables	mrTRG		*P* value	pTRG		*P* value
	mrTRG 0–1	mrTRG 2–3		pTRG 0–1	pTRG 2–3	
The maximum short-axis of LLN before nCRT(mm)	8.54 ± 4.85	8.14 ± 3.75	0.661^γ^	8.07 ± 4.45	8.47 ± 3.95	0.648
The maximum short-axis of LLN after nCRT (mm)	5.25 ± 4.13	5.48 ± 3.81	0.791^γ^	4.79 ± 3.81	5.90 ± 3.95	0.176
Short-axis reduction rate (%)	43.1 (1.0–82.4)	39.7 (−11.4–73.8)	0.493^δ^	42.4 (1.0–82.4)	31.4 (−11.4–73.8)	0.049^δ^

*LLN* Lateral lymph node; *nCRT* Neoadjuvant chemoradiotherapy; *mrTRG*: Magnetic resonance imaging tumor regression grading; *pTRG* Pathological tumor regression grading

^γ^t test

^δ^Wilcoxon test

## Discussion

Previous studies suggested that the probability of LLNM in advanced rectal cancer was 10%-25% [29, 30]. LLNM also is an independent risk factor for poor prognosis in advanced rectal cancer [31]. Therefore, LLN should not be ignored in the treatment of advanced rectal cancer. The incidence of LLNM in patients with suspected positive LLN before operation was 8.1%-51.6% after nCRT [8, 24, 32]. The incidence in this study was 26.4%. It demonstrated that nCRT with TME alone was not enough to ensure the R0 resection of such patients. Meanwhile enlarged LLN is a high risk factor for LLR [33, 34]. Therefore, TME plus sLLND after nCRT for such diseases is necessary. However, which patients should be performed LLND is always discussed, but which patients should be exempted from LLND is rarely mentioned in clinic.

Previous studies suggested the maximum short-axis was closely related to LLNM [8, 23, 24], and chose 8 mm before nCRT [23, 24] and 5 mm after nCRT [8, 22] as the cut-off values. The cut-off values in this study also obtained the similar results, whether it was the maximum short-axis before and after nCRT or the cut-off values of the maximum short-axis before and after nCRT, there were significant differences between the two groups.

At present, increasing numbers of studies suggested that the comprehensive treatment strategy of combined nCRT and sLLND is for advanced rectal cancer patients with suspected LLNM based on imaging findings [7, 8, 35]. Accurate assessment and application of TRG was not only of great significance for predicting the prognosis of patients with advanced rectal cancer, but also for guiding the "watch and wait" strategy [36]. In previous reports, mrTRG system developed by MERCURY study identified a favorable outcome subgroup with extended OS and disease-free survival rate (DFS) [37, 38]. It was similar to pTRG principle, however, whether that mrTRG system could be used as a substitute for pTRG system remains controversial due to the poor consistency with pTRG [39–41].

Fortunately, a new four-category mrTRG system developed by Pang et al.[16] achieved good agreement with pTRG (к = 0.671) while achieving good efficiency in identifying complete pathological responses. Therefore, we chose this system to evaluate the mrTRG of our patients. According to our results, the consistence between mrTRG and pTRG was good agreement (к = 0.684). It demonstrated that the understanding of this mrTRG system was correct and the results were reliable. Multivariate logistic regression showed that mrTRG and pTRG were the independent predictors of LLNM, respectively. Sun et al. [10] found that mesorectal LRG was basically consistent with pTRG. Although we did not assess lateral LRG, our results showed that the LLNM and TRG also remained highly consistent no matter which system of TRG was chosen. Only using mrTRG to predict LLNM achieved the sensitivity of 0.875 and NPV of 0.909. Only using pTRG to predict LLNM achieved the basically same results (sensitivity of 0.875, NPV of 0.927). The results of subgroup analysis also showed that the short-axis reduction rate in pTRG 0–1 was significantly higher than that in pTRG 2–3. It illustrated that the sensitivity of LLN to nCRT was identical to primary tumor. In addition, under the condition of good agreement between mrTRG and pTRG, different results were found when mrTRG and pTRG were used to analyze the short-axis reduction rate, which may be related to the small number of cases in this study, and small data changes could affect the final results. The length diameter reduction rate of primary tumor is related to TRG, so there are differences between groups and in univariate analysis, but it is meaningless in multivariate analysis. I wonder if this is related to our small sample size.

The combination of mrTRG, the maximum short-axis of LLNs ≥ 8 mm before nCRT and the maximum short-axis of LLN ≥ 5 mm after nCRT was named to Risk score A, which achieved satisfactory specificity (0.970), acceptable PPV (0.867) and NPV (0.855). The same combination used by pTRG was named Risk score B, which also achieved the satisfactory specificity (0.970), acceptable PPV (0.857) and NPV (0.844). These two risk scores show their good sensitivity to true negative LLN, which had important guiding significance for sLLND. Although it could not help us accurately determine which patient should undergo sLLND, it told us when physical examination, laboratory tests and imaging examinations indicated that the primary tumor and the suspected positive LLN all obtained good regression after nCRT, we should be cautious in performing LLND. Therefore, accurate assessment of the regression of primary tumor and suspected positive LLN could avoid unnecessary LLND. At the same time, because the East and the West have not reached an agreement on the treatment strategy of patients with suspected LLNM, there must be many patients who are indicative of sLLND might not have received it. Our results also had positive guiding significance for further treatment of this part of patients. The accurate pTRG and the changes in the maximum short-axis of suspected positive LLN before and after nCRT could tell us which patient should be safe to choose "watch and wait".

Different to previous studies, our results revealed that the positive rate of LLNs was not related to the location [42]. The possible reason may be that we only analyzed the LLNs with maximum short-axis. Previous studies revealed that age, female, low rectal cancer, EMVI positive, MRF positive, CEA were not statistically significant related to LLNs status [43],which were similar to our results.

The shortcomings of this study were as follows: firstly, the number of cases was relatively small, which was not enough to establish a prediction model. Secondly, it was impossible to analyze the relationship between clinical complete regression and LLNM because the colonoscopy data before nCRT of many patients were unavailable. Thirdly, because most patients had a short follow-up period (less than 2 years), we lacked the analysis of long-term outcome for these patients. However, this result can be utilized to avoid an unnecessary LLND when the responses of primary tumor and LLNs to nCRT are both good.

## Conclusion

The suspected positive LLNs tend to be sterilized by nCRT in patients who have a very good response to nCRT. This study suggests that it is rational to avoid LLND in patients whose primary tumor and LLNs both show good response to nCRT.

## Supplementary Information

**Additional file 1: Table S1.** Evaluation criteria of MRF. **Table S2.** Evaluation criteria of EMVI. **Table S3.** Evaluation criteria of MRI-positive LLN before and after nCRT. **Figure S1.** The method for determining the correspondence between the LLN and the pathological outcomes. The scissors point to the LLN with the maximum short-axis.

## Data Availability

The datasets used and/or analyzed during the current study are available from the corresponding author on reasonable request.
