# Supplementary material for: Can patients with good tumor regression grading after neoadjuvant chemoradiotherapy be exempted from lateral lymph node dissection?
Source: Discov Oncol. 2022 Dec 30;13:144. doi: 10.1007/s12672-022-00607-w (PMC9800664; doi:10.1007/s12672-022-00607-w)
Supplement: Supplementary file 1 — Additional file 1: Table S1. Evaluation criteria of MRF. Table S2. Evaluation criteria of EMVI. Table S3. Evaluation criteria of MRI-positive LLN before and after nCRT. Figure S1. The method for determining the correspondence between the LLN and the pathological outcomes. The scissors point to the LLN with the maximum short-axis. [file 12672_2022_607_MOESM1_ESM.docx]

Table S1. Evaluation criteria of MRF

| MRF | Description |
| --- | --- |
| Positive | Diffuse iso- or relatively high-intensity infiltration of MRF on T2-WI or diffuse low-intensity tissue infiltration with a distance ≤2 mm between tumor and MRF. |
| Negative | Otherwise. |

MRF: mesoretal fascia

Table S2. Evaluation criteria of EMVI

| EMVI | Description |
| --- | --- |
| 0 score | Tumor extension is not nodular and no adjacent vessels. |
| 1 score | Minimal extramural stranding and no adjacent vessels. |
| 2 score | Retention near blood vessels and no tumor signal in normal caliber lumen. |
| 3 score | Moderate signal within vessel lumen and dilation of small vessels. |
| 4 score | Irregular vessel contour and definite tumor signal. |

EMVI: extramural venous invasion; 0-2 was defined as negative and 3-4 as positive.

Table S3. Evaluation criteria of MRI-positive LLN before and after nCRT

| Malignant features of LLN in MRI | Round, irregular borders and heterogeneity. |
| --- | --- |
| MRI-positive LLN criteria before nCRT | LLN with the maximum short-axis <5 mm need three malignant features, LLN with the maximum short-axis of 5～8 mm need two malignant features, and LLN with the maximum short-axis ≥9 mm were directly judged as positive. |
| MRI-positive LLN criteria after nCRT | The maximum short-axis ≥5mm. |

Figure S1 The method for determining the correspondence between the LLN and the pathological outcomes.


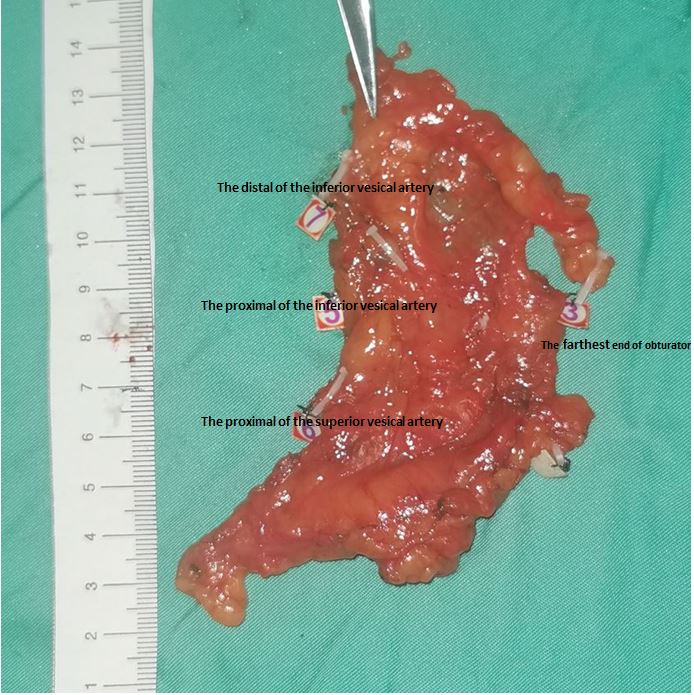


The scissors point to the LLN with the maximum short-axis.
